# Evaluating the impact of the DREAMS partnership to reduce HIV incidence among adolescent girls and young women in four settings: a study protocol

**DOI:** 10.1186/s12889-018-5789-7

**Published:** 2018-07-25

**Authors:** Isolde Birdthistle, Susan B. Schaffnit, Daniel Kwaro, Maryam Shahmanesh, Abdhalah Ziraba, Caroline W. Kabiru, Penelope Phillips-Howard, Natsayi Chimbindi, Kenneth Ondeng’e, Annabelle Gourlay, Frances M. Cowan, James R. Hargreaves, Bernadette Hensen, Tarisai Chiyaka, Judith R. Glynn, Sian Floyd

**Affiliations:** 1 Faculty of Epidemiology and Population Health, London School of Hygiene & Tropical Medicine, Keppel Street, London, WC1E 7HT UK; 2 University of California at Santa Barbara, Santa Barbara, USA; 3 Centre for Global Health Research, Kenyan Medical Research Institute, Kisumu, 40100 Kenya; 4 Institute for Global Health, University College of London, Capper St, London, WC1E 6JB UK; 5 Africa Health Research Institute, 719 Umbilo Road, Durban, KwaZulu-Natal 4001 South Africa; 6 African Population and Health Research Center, Manga Close off Kirawa Road, Nairobi, Kenya; 7 Department of Clinical Sciences, Liverpool School of Tropical Medicine, Pembroke Place, Liverpool, L3 5QA UK; 8 Department of International Public Health, Liverpool School of Tropical Medicine, Pembroke Place, Liverpool, L3 5QA Zimbabwe; 9 Centre for Sexual Health and HIV/AIDS Research (CeSHHAR), 9 Monmouth Road, Avondale West Harare, Zimbabwe; 10 Faculty of Public Health & Policy, London School of Hygiene & Tropical Medicine, 15-17 Tavistock Place, London, WC1H 9SH UK; 11 Faculty of Infectious and Tropical Diseases, London School of Hygiene & Tropical Medicine, Keppel Street, London, WC1E 7HT UK

**Keywords:** HIV prevention, Adolescent health, Complex intervention, Impact evaluation, Gender equity, Kenya, South Africa, Zimbabwe

## Abstract

**Background:**

HIV risk remains unacceptably high among adolescent girls and young women (AGYW) in southern and eastern Africa, reflecting structural and social inequities that drive new infections. In 2015, PEPFAR (the United States President’s Emergency Plan for AIDS Relief) with private-sector partners launched the DREAMS Partnership, an ambitious package of interventions in 10 sub-Saharan African countries. DREAMS aims to reduce HIV incidence by 40% among AGYW over two years by addressing multiple causes of AGYW vulnerability. This protocol outlines an impact evaluation of DREAMS in four settings.

**Methods:**

To achieve an impact evaluation that is credible and timely, we describe a mix of methods that build on longitudinal data available in existing surveillance sites prior to DREAMS roll-out. In three long-running surveillance sites (in rural and urban Kenya and rural South Africa), the evaluation will measure: (1) population-level changes over time in HIV incidence and socio-economic, behavioural and health outcomes among AGYW and young men (before, during, after DREAMS); and (2) causal pathways linking uptake of DREAMS interventions to ‘mediators’ of change such as empowerment, through to behavioural and health outcomes, using nested cohort studies with samples of ~ 1000–1500 AGYW selected randomly from the general population and followed for two years. In Zimbabwe, where DREAMS includes an offer of pre-exposure HIV prophylaxis (PrEP), cohorts of young women who sell sex will be followed for two years to measure the impact of ‘DREAMS+PrEP’ on HIV incidence among young women at highest risk of HIV. In all four settings, process evaluation and qualitative studies will monitor the delivery and context of DREAMS implementation. The primary evaluation outcome is HIV incidence, and secondary outcomes include indicators of sexual behavior change, and social and biological protection.

**Discussion:**

DREAMS is, to date, the most ambitious effort to scale-up combinations or ‘packages’ of multi-sectoral interventions for HIV prevention. Evidence of its effectiveness in reducing HIV incidence among AGYW, and demonstrating which aspects of the lives of AGYW were changed, will offer valuable lessons for replication.

**Electronic supplementary material:**

The online version of this article (10.1186/s12889-018-5789-7) contains supplementary material, which is available to authorized users.

## Background

The incidence of HIV is declining or stabilizing in many settings, yet levels of new infections remain unacceptably high among adolescent girls and young women (AGYW) [1]. In almost all countries with generalized epidemics, young women aged 15–24 years are three to five times more likely than their male counterparts to be living with HIV; and in sub-Saharan Africa, 71% of new infections in adolescents are among girls [1]. In a pattern that is consistent across most high prevalence countries, HIV incidence rates rise dramatically between the ages of 15 and 24, and more steeply among females than males [2].

As the world’s population of adolescents grows, particularly in east and southern Africa, high incidence among young people will equate to rises in the absolute numbers of new infections [2, 3]. The role of adolescent HIV prevention in broader epidemic control is recognized with the growing commitment at global and national levels to prioritise young people in efforts to end the AIDS epidemic. With the ‘All In to End Adolescent AIDS’ campaign, for example, UNICEF and global partners seek to reduce new HIV infections among adolescents (10–19 years) by 75% between 2015 and 2020, and ‘end’ the AIDS epidemic among adolescents by 2030 (to fewer than 200 infections per year) [3]. The complexity of this goal is not underestimated, and the multidimensional nature of AGYW vulnerability has to date proven resistant to change by single interventions, sectors or disciplines [4]. The need for combination approaches, and ‘packages’ of interventions, is increasingly recognised. For example, the recent issue of *Disease Control Priorities* recommends an essential and cost-efficient ‘package’ to be delivered in adolescence – through a mixed approach involving the community, media and health systems [5]. Similarly, a ‘call for action’ on HIV prevention, ‘HIV Prevention 2020’, specifies a combination of primary prevention interventions, to be designed comprehensively and delivered effectively and at scale among populations at greatest risk [6].

The ‘DREAMS’ Partnership (http://www.dreamspartnership.org/) is an ambitious programme aiming to halt AGYW infections through such an approach: a broad package of evidence-based health, educational and social interventions to be delivered with urgency, high coverage, and where the need is greatest. On World AIDS Day 2014, the United States President’s Emergency Plan for AIDS Relief (PEPFAR), the Bill & Melinda Gates Foundation and the Nike Foundation announced the DREAMS investment in 10 countries in sub-Saharan Africa [7]. The goal of DREAMS is to reduce new infections by 40% after two years of intervention among AGYW in sub-national geographic units identified as ‘hot-spots’ with high HIV burden.

By investing in a multi-component package, DREAMS aims to address the root causes of girls’ and young women’s vulnerability and improve their lives more broadly – their value in society and their own esteem, their experiences within relationships, opportunities for schooling and employment, and healthy transitions from adolescence to adulthood. The Partnership aims to ensure that AGYW have an opportunity to live *D*etermined, *R*esilient, *E*mpowered, *A*IDS-free, *M*entored and *S*afe lives (‘DREAMS’) in high-burden settings, through interventions targeting young women, their families, community and male sexual partners [7].

Evidence of DREAMS’ effectiveness can stimulate a renewed focus on HIV prevention [6]. We sought the best opportunities to independently evaluate the impact of DREAMS in selected settings, in both general and key population groups, to offer lessons to those implementing DREAMS and to inform future investments in young women’s health and well-being. To maximize the potential for generating evidence around DREAMS, four diverse settings in three countries – Kenya, Zimbabwe, and South Africa – were chosen for this evaluation, based on the availability of existing demographic and HIV data platforms that would enable credible and timely evaluation. The diversity of settings is an asset to the generalisability of this evaluation, with each site presenting distinct opportunities to generate evidence, but it raises challenges in terms of ensuring comparability and an appropriate level of harmonization across the different settings. This paper presents the overall protocol for the evaluation in all four sites; details of the design unique to the evaluation in Zimbabwe are published elsewhere [8] and site-specific protocols for the other three settings are available upon request.

The impact evaluation is funded independently of DREAMS’ implementation and is a collaboration between the London School of Hygiene & Tropical Medicine (LSHTM, UK); the Africa Health Research Institute (AHRI) in KwaZulu-Natal, South Africa; the African Population and Health Research Center (APHRC) in Nairobi, Kenya; the Centre for Sexual Health and HIV AIDS Research (CeSHHAR) in Zimbabwe; the Kenya Medical Research Institute (KEMRI) in Kisumu, Kenya; and the Liverpool School of Tropical Medicine (LSTM, UK).

### The DREAMS core package

The DREAMS Partnership supports a core package of interventions targeted at AGYW, their families, wider communities, and men characterized to be the sexual partners of AGYW [7]. The package is comprised of evidence-based interventions shown to address HIV risk behaviours, HIV transmission, socio-economic vulnerabilities and gender-based violence (Table 1).

**Table 1:** Interventions and target populations of the DREAMS Core Package

Target population and strategy	Evidence-based intervention
*Individual interventions (delivered directly to adolescent girls and young women)*	
Empower girls and young women and reduce their risk	▪ Condom promotion and provision ▪ HIV testing and counselling services (HTS) ▪ Oral pre-exposure prophylaxis (PrEP) for HIV, offered to a subset of females at exceptionally high risk and in select countries ▪ Post-violence care ▪ Expanded contraceptive method mix ▪ Social asset building
*Contextual interventions (not all delivered directly to adolescent girls and young women but from which they can benefit)*	
Mobilize and strengthen the community for change	▪ School-based HIV and violence prevention for boys and girls ▪ Community-based HIV and violence prevention for boys/young men and girls/young women ▪ Community mobilization and norms change for community leaders, boys and men
Strengthen families	▪ Parenting and caregiver programmes for vulnerable adolescent girls ▪ Social protection (cash transfers, educational subsidy, combination socioeconomic approaches)
Decrease risk in sexual partners of AGYW	▪ Characterisation of male partners to target highly effective interventions, e.g., HIV testing services, antiretroviral therapy (ART) and voluntary medical male circumcision (VMMC)

DREAMS investments aim to ensure that AGYW in selected DREAMS areas (sub-national units with high HIV burden) have access to all core package interventions, either through DREAMS funding or additional PEFPAR funding schemes (e.g., for VMMC) or coordination with national government programmes (e.g., for cash transfers or educational subsidies). PrEP is planned for selected countries and sites within countries, as determined by national governments. Guidance for each component of the core package has been provided to countries by PEPFAR, and coverage targets have been set for each sub-national unit by age group, area and intervention [9]. ‘Primary’ interventions are the priority interventions from the core package that all AGYW in an age group should receive. ‘Secondary’ interventions are needs-based interventions from the core package, recommended for specific sub-populations of AGYW based on additional circumstances, e.g., condom provision for AGYW who are sexually active; post-violence care for those who have experienced sexual violence. Additional file 1: Table S1 summarises the primary and secondary interventions in each country setting.

The way in which the various DREAMS components are rolled out and coordinated, and the timing of implementation will differ in each evaluation site, depending upon: the capacity and readiness of Implementing Partners (IPs) contracted by the United States Government to implement DREAMS services; the timing of contractual arrangements with IPs; negotiations with national governments; finalization of sex education curricula for schools; and other contextual factors. Given the heterogeneity in DREAMS’ delivery, we will monitor how, when, by whom, and to whom, components of the DREAMS package are delivered, in the process evaluation activities described below [10].

## Methods/Design

### Aims & objectives

This protocol outlines the plans to evaluate the impact of the DREAMS programme at the individual and population levels in four sub-Saharan African settings representing diverse epidemiological and social contexts. The evaluation aims to answer three main questions:

1. What is the impact of the combined DREAMS package on HIV infection rates and other key outcomes, among AGYW and men who are in the age range that includes most sexual partners of AGYW?
2. Through what pathways do DREAMS interventions affect the health, education, and social well-being of individual AGYW?
3. What interventions were implemented and how (with what timing, coverage and quality)?

In the South African and two Kenyan sites, the impact of DREAMS, including community, facility, and school-based interventions, on HIV infection rates and other key outcomes will be measured in the general population. In Zimbabwe, the impact of a combination DREAMS package which includes an offer of oral PrEP, alongside other interventions, on HIV infection rates and other key outcomes will be evaluated among young women who sell sex (YWSS) [8].

### Theory of change

**Fig. 1:**
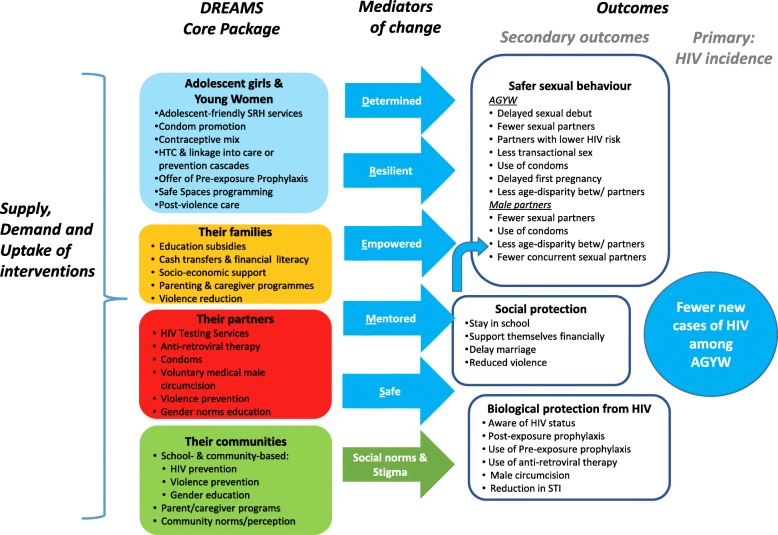
Theory of change to guide the impact evaluation

We hypothesize that DREAMS will reduce incidence of HIV among AGYW through three related pathways of protection (Fig. 1):

1. Social Protection: DREAMS will reduce social and economic vulnerability of AGYW by helping them to stay in school; enabling financial independence to offer socioeconomic alternatives to early marriage and transactional sex; and reducing gender-based violence and financial dependence on intimate partners.
2. Sexual Behavior: DREAMS will reduce acquisition of HIV by promoting safer sexual behaviors and sexual networks among AGYW and their male partners, including through increased condom use.
3. Biological Protection: DREAMS will reduce the likelihood of AGYW acquiring HIV through biomedical technologies that lower the risk of transmission of the virus (by reducing viral load among HIV-positive male partners, through increased knowledge of HIV status and uptake of ART), and reducing the risk of acquisition of HIV (PrEP, and VMMC for male partners).

Psycho-social mediators of change, such as empowerment and self-efficacy, are hypothesised to link uptake of DREAMS interventions by AGYW to the three pathways of protection and ultimately to a reduction of HIV incidence among AGYW.

The impact of DREAMS interventions will depend on the scale and intensity at which they are delivered and whether they are accessed. Through the process evaluation, we will assess the roles of supply of, demand for, and adherence to, DREAMS interventions, as per the conceptual framework for HIV prevention cascades [11]. More specifically, we will investigate the extent to which interventions in the core package work in combination to enhance the supply of prevention products/programmes, to limit barriers to access, and to create and enhance opportunities and motivations for AGYW and young men to adopt and adhere to them [10, 12].

### Study settings

To maximize the potential for generating evidence on the impact of the DREAMS Partnership, four settings in three countries were chosen, each with existing demographic and/or HIV data platforms. In the South African and two Kenyan sites, this evaluation will make use of long-standing longitudinal health and demographic surveillance systems (HDSS), while in Zimbabwe a national programme to provide HIV-related services to sex workers will serve as the evaluation’s starting point. The HDSS provide direct measurement of trends in HIV incidence as well as demographic, sexual behavior, and linked clinical data to evaluate DREAMS’ impact. Data from the national programme for sex workers in Zimbabwe provide estimates of past HIV incidence and a platform from which to identify and reach AGYW at highest risk of HIV.

#### uMkhanyakude, KwaZulu-Natal, South Africa

The Africa Health Research Institute (AHRI; formerly the Africa Centre for Population Health) in uMkhanyakude, KwaZulu-Natal, has followed a total of ~ 160,000 individuals from 11,000 geocoded households from 2000, in a 428 km^2^ surveillance area. Demographic surveys have been conducted three times a year, with annual collection of individual socio-economic, behavioral, and HIV service uptake data alongside collection of dried blood spots for laboratory testing for HIV infection. AHRI has a memorandum of understanding with the Department of Health that enables linkage of the population surveillance data to the primary care electronic record systems in the local health care facilities (2010 onwards) and to the TIER.Net electronic record system for HIV treatment (2004 onwards), as well as to all clinical laboratory test results of patients in the sub-district through linkage with the National Health Laboratory Systems (NHLS) database (since 2004). Since 2017, AHRI has embedded clinical research assistants in all primary health care settings in the surveillance area. They electronically capture details on the reason for attendance, and these clinic attendance data are linked to the demographic surveillance data.

#### Gem sub-county, Siaya county, Kenya

The Kenya Medical Research Institute (KEMRI)/Centers for Disease Control and Prevention (CDC) HDSS site in Siaya County of western Kenya covers a total population of approximately 223,000 people and 55,000 households, with demographic surveys three times a year. Siaya County includes three sub-counties: Rarieda, Siaya, and Gem. For the evaluation of DREAMS, the KEMRI/CDC platform in Gem will be used, as this is where HIV surveillance has been conducted most frequently and recently, i.e., three behavioral surveys and four rounds offering HIV testing services in 2011/2012, 2013/14, 2016, and 2017, among a random sample of one-quarter of all households and all resident members of those households as an open cohort [13].

#### Nairobi county, Kenya

The African Population and Health Research Center (APHRC) began the first urban-based longitudinal HDSS platform in sub-Saharan Africa, known as the Nairobi Urban Health and Demographic Surveillance System (NUHDSS) in 2002 in two informal slum settlements of Nairobi: Korogocho and Viwandani [14]. The NUHDSS covers approximately 65,000 people and 24,000 households in 14 villages with quarterly sociodemographic surveys and annual surveys (2012–2016) on fertility preferences. As the last HIV serological survey was conducted in 2007, HIV incidence will not be measured in this setting. This site is conducting formative research with 10–14 year olds for the Global Early Adolescent Study (GEAS) [15], and will therefore be able to include impact evaluation analyses from age 10 (unlike the other 3 evaluation settings which will focus on AGYW from age 13).

#### Zimbabwe

The Zimbabwe evaluation will capitalise on a national programme that provides HIV prevention and sexual and reproductive health services to female sex workers (FSW) in Zimbabwe, known as “Sisters with a Voice”. ‘Sisters’ began in 2009 and provides free access to HIV testing, STI treatment, family planning, HIV prevention education, condoms and legal services to over 65,000 women across 36 sites [16]. Around 40% of FSW accessing the programme are younger than 25 years. The evaluation will include six districts in which the Sisters programme is active: two in which DREAMS+PrEP are delivered (Bulawayo and Mutare) and four comparison sites in which no DREAMS interventions are planned (Karoi, Chinhoyi, Zvishavane and Kwekwe). Comparison sites were selected for their comparability to intervention sites in terms of population size, urban location, and presence of a Sisters with a Voice site with relatively high client volume [8].

### Study design

The DREAMS package of interventions prioritises AGYW, but also includes ‘contextual’ components directed at young men, the families of AGYW, and the wider community (Table 1). Consequently, the overall impact of DREAMS interventions should be measured at community, or “population”, level. For example, if HIV incidence among AGYW is reduced following DREAMS interventions then this is likely to have been achieved through increased uptake of services and behaviour change among men as well as among AGYW themselves.

In the evaluation settings in which impact is measured in the general population (Nairobi, Gem, and Umkhanyakude), the primary way in which the impact of the DREAMS programme will be measured is through comparisons of HIV incidence (Gem and Umkhanyakude only), and HIV-related outcomes (in all 3 settings) across calendar time periods before, during early roll-out, and after DREAMS programmes have been established. This has the disadvantage that changes over time may be due to factors other than DREAMS interventions, but the advantage is that comparisons are made within the same setting and population, at multiple time points. A cluster-randomised trial design was not possible because the priority of the DREAMS Partnership was for rapid roll-out of DREAMS investments to geographic areas specifically chosen for their relatively high HIV prevalence, rather than to a randomly selected sample of areas [9].

For additional evidence of plausibility and impact, changes in outcomes will be assessed by estimating dose-response relationships between DREAMS uptake and outcomes at the small-area level [17]. ‘Layering’ of multiple interventions or services from the DREAMS core package, through integration and referrals, will be a key way of quantifying dose, for example, as the percentage of AGYW who received multiple intervention components and/or the minimum package designed for their age.

In Zimbabwe, the main way in which impact will be measured is through a comparison between 2 districts which will receive DREAMS interventions with 4 districts that will not. This alternative study design was chosen because the study population is young women who sell sex (not the general population of AGYW), who are at high risk of HIV acquisition in all 6 study districts, and a respondent-driven sample of YWSS will be enrolled into a cohort study and followed up for two years [8].

As well as measuring the overall impact of DREAMS interventions at population-level, in the Kenyan and South African settings a random sample of AGYW will be enrolled into a “nested” (within the total population) cohort study and followed up for two years, in order to collect more detailed data on awareness and uptake of interventions, psycho-social “mediators of change”, and the three hypothesized pathways of change (social protection, sexual behaviour, and biological protection), and thus enable in-depth analysis of pathways of change.

**Fig. 2:**
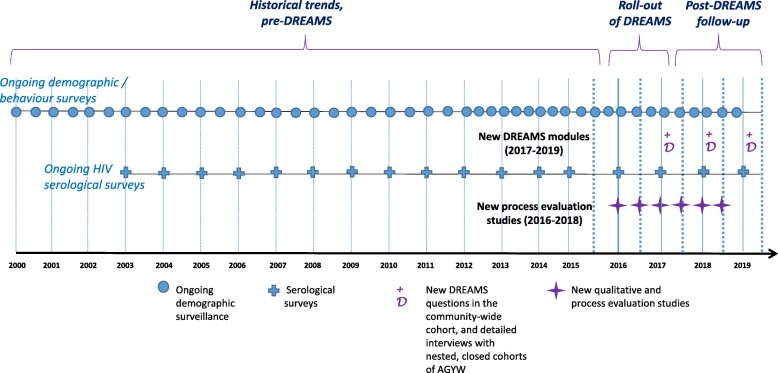
Timing and components (ongoing and new) of data collection embedded within a population platform: example of uMkhanyakude, South Africa

To achieve the above, the design comprises three main components:

1. **Population-based surveillance systems**: In uMkhanyakude, and Gem and Nairobi in Kenya, existing surveillance systems that link HIV, demographic, behavioural, and service uptake data, will be used and enhanced in order to assess the population-level effects of DREAMS over time (in relation to the timing of DREAMS roll-out) among AGYW, men who are in the age range that includes most of the partners of AGYW, and also older adults who may receive DREAMS interventions that are directed at the wider community. In uMkhanyakude and Gem, linkage to HIV clinic data is possible and geospatial data are available, and in uMkhanyakude HIV phylogenetics data are also available. We will utilise historical (for baseline) and prospective data (for comparison) from the population-based systems (see Fig. 2 for example in South Africa).
2. **Cohorts of AGYW**, randomly selected from the total population: For detailed study of the pathways by which DREAMS interventions influence HIV risk, we will establish cohorts of AGYW within each evaluation site. Cohort enrolment will be completed during the early roll-out of DREAMS interventions, during 2017, and those enrolled will be followed prospectively, at ~ 12 and ~ 24 months later. There will be more detailed and comprehensive data collection on uptake of DREAMS interventions, mediators of change, and socio-economic, behavioural and health outcomes than is possible in the total population. In the Zimbabwe setting, the same cohort used to measure the overall impact of DREAMS interventions on HIV incidence among YWSS can be used to analyse pathways of change.
  In uMkhanyakude, Nairobi, and Gem, nested cohorts of AGYW aged 13–22 will be selected using the HDSS census population as the sampling frame. A random sample of AGYW stratified by age (13–17 and 18–22 years) and area of residence will be selected. The Nairobi evaluation will further recruit a sample of young girls from age 10 (building on the Global Early Adolescent Study pilot in this setting), resulting in three age groups for the cohorts: 10–14, 15–17, and 18–22 years.

In Zimbabwe, the network-based recruitment strategy used to identify and refer YWSS to the DREAMS (intervention sites) or Sisters (in comparison sites) programme, is described in detail elsewhere [8]. This recruitment strategy is appropriate in the absence of a sampling frame and when the population of interest is primarily hidden as is the case among young women who sell sex in Zimbabwe.

3) **Process evaluation**: In all four DREAMS evaluation sites, a process evaluation will use both qualitative and quantitative methods to describe DREAMS implementation in context, and to challenge and interrogate causal assumptions in the theory of change [18]. To understand DREAMS’ influence on supply, demand, and uptake of interventions (the ‘prevention cascade’) [12], we will investigate reach and coverage, views and experiences of DREAMS components, what helps and hinders successful implementation and uptake, and to what extent implementation is influenced by differing social and epidemiological contexts.
  Specifically, we will explore fidelity (whether all components of DREAMS were implemented on schedule and as planned), feasibility (identifying barriers and facilitators to implementation), acceptability (how staff and beneficiaries perceive and value the intervention), and quality (measured by both objective and subjective criteria). In the process, we will aim to identify unexpected pathways and consequences, and who is left out (equity).

The process evaluation will include five methodologies:

- Qualitative longitudinal study of young people’s experiences. Young people’s experience of and “journeys” through DREAMS, including barriers and facilitators to what works in practice will be tracked in detail for a small cohort of 20 AGYW and, in HDSS sites only, 20 young men in each site. These cohorts – of DREAMS beneficiaries sampled purposively from the general population (for males) and AGYW cohorts (for females) – will be followed longitudinally and offered a range of ways to share their experiences in real-time including use of diaries and informal interviews.
- Small group discussions. The experiences of AGYW’s families, parents, partners, and broader communities will be explored through focus and family group discussions. Group discussions in each evaluation setting will help investigate understanding and experience of DREAMS and its components and whether social norms and attitudes are influenced by the interventions.
- Rapid participatory community mapping. This method will be used in the DREAMS areas to quickly gain a broad understanding of the social context for adolescents and young people and the reach and coverage of the AGYW services at baseline and after two years of DREAMS intervention. The mapping will use rapid appraisal methods with participant observation and short interviews.
- Interviews with key informants in delivery organizations. Up to 20 individuals responsible for implementing DREAMS activities in each setting will be interviewed to explore views and experiences of, and barriers and facilitators to, DREAMS activities each year. Local health care workers and community and youth leaders will also be interviewed.
- Observations of DREAMS interventions delivered in context. Using checklists, structured observations will record the ways in which DREAMS is delivered and received, and with what quality and intensity (using DREAMS standard operating procedures for reference). Observations will be made of DREAMS interventions in a range of settings such as schools, safe spaces, and health facilities.

### Measurement and analysis of key variables

For component 1, the population-based surveillance in the general population of AGYW and men, the primary comparison is across three time periods: pre-DREAMS, during the early roll-out of DREAMS interventions, and post-DREAMS. The aim is to know whether HIV incidence among AGYW aged 15–24 years (the primary outcome, directly observed through repeat testing) and key secondary outcomes, measured among both AGYW and men, have changed over time at population-level. The primary and secondary outcomes are summarized in Table 2, with secondary outcomes lying on the three pathways of central interest that are between the interventions and HIV incidence: social, behavioural (sexual), and biological protection. The extent to which any changes can be attributed to DREAMS interventions will be assessed in the context of other secular changes, and the findings of the process evaluation. For example, given the background scale-up of universal testing and treatment for HIV, our findings on HIV incidence trends among AGYW will be placed in the context of trends in HIV incidence and the uptake of HIV testing and treatment among those who are not directly targeted for DREAMS HIV prevention interventions.

**Table 2:** Primary and secondary measures of impact at the population-level in the South African and Kenyan evaluation sites, to be estimated by comparing calendar time periods that represent pre-DREAMS, during early roll-out of DREAMS, and after DREAMS roll-out

	Adolescent girls and young women		Male sexual partners
	15–19 years	20–24 years	(15–34 years)
Primary Outcome	HIV incidence^a^		HIV incidence^a^
Secondary Outcomes			
*Biological protection*	Knows HIV status^b^		
	HIV/STI prevalence	STI prevalence & Incidence	Uptake of VMMC
			Use of ART
			Community HIV viral load^d^
*Behavioral protection*	Ever had sex		Number of sexual partners (in last year / lifetime)
	Age at first sex		
	Number of sexual partners (in last year / lifetime)		
	Age-disparity with sexual partners		
	Ever been pregnant	Unmet need for contraception^c^	Concurrency of sexual partners
	Age at first / subsequent pregnancies		
	Use of condoms^e^ Any condomless sex		
	Transactional sex		
*Social protection*	In or completed school	In employment or completed vocational / microfinance training	Gender norms: support gender equity
	Age at first marriage		Experience of violence (exposure to / victimization / perpetration)
	Experience of violence		

^a^not collected in Nairobi

^b^know they are HIV+ or have tested HIV negative in the past 12 months

^c^ do not want a child in next 2 years or ever but not using a method to prevent pregnancy

^d^ uMkhanyakude only

^e^ Used condom at last sex (in past 12 months); Any condomless sex in last 1 month / last 12 months and in the last 3 months

For component 2, the nested cohorts of AGYW designed to measure pathways of change, the primary exposure is uptake of DREAMS interventions among individual AGYW, considering single components as well as the number and combination of components of the core package that were received. The extent to which AGYW are aware of, invited into, and participate in DREAMS interventions will be summarized, using the core package and primary/secondary interventions as frameworks to categorise interventions and standardize across the settings. (See Table 3 for proposed, a priori measures of DREAMS uptake.) Comparisons of mediators and secondary outcomes will then be made among AGYW according to their uptake of DREAMS interventions.

**Table 3:** Outcomes, mediators of change in the outcomes, and uptake of DREAMS interventions, to be captured via nested DREAMS cohorts of adolescent girls and young women (in Kenya and South Africa) and young women who sell sex (in Zimbabwe)

	AGYW (South Africa and Kenya)		YWSS (Zimbabwe)
	13–17 years^b^	18–22 years^b^	18–24 years
Primary	n/a ^a^		HIV incidence
Secondary Outcomes	Knows HIV status		
	Number of lifetime pregnancies		
	Reduced experience of violence		
	Incidence & prevalence of HSV-2/other STI		HIV prevalence
	Aware of partners’ HIV status		Number of sexual partners
			Engaged in transactional sex for economic reasons
	Age at first sex	Number of sexual partners in the last 12 months	Use of condoms & PrEP with regular/transactional sex partners
	Age at first /subsequent pregnancies	Age at first / subsequent pregnancies	Adherence to HIV treatment and care services
	Condom use		Reduced food insecurity
	Unmet need for contraception		
	Age-disparity with sexual partners	HIV risk of sexual partners (to be defined a priori)	
	No/less transactional sex		
	Stay in school	In employment or completed vocational / microfinance training	
	Age at first marriage or first long-term/live-in partner		
Mediators of change (Sample measures of constructs that DREAMS aims to improve, specifically: social assets, personal safety, self-efficacy, common mental disorders, empowerment, gender equitable norms, and sexual relationship power)	Have at least one trusted female friend they can confide in		Have at least one trusted female friend they can confide in
	Meet regularly in a safe place with peers		Meet regularly at community mobilization sessions with peers and has increased sense of social cohesion
	Know a woman, other than mother/guardian, to turn to if have a serious problem		Can access HIV prevention services including condoms, STI treatment & PrEP (as measure of self-efficacy) and contraceptives
			Is supported to adhere to PrEP, and economically able to adhere/access PrEP
	Able to avoid / refuse sex if sex is not wanted		Has comprehensive knowledge of HIV prevention
	Able to refuse sex if partner will not use a condom (or confident they can use a condom with all sex partners)		Have access to money in an emergency
	Confident they could get a HIV test		Is confident she can negotiate condom use with sexual partners (including clients)
	Confident they can access health services when they need them (sexual and reproductive health services in particular)		Able to avoid violent relationships
	Have access to their own savings		
	Have access to money in an emergency		
	Believes a man and woman should decide together whether to use a condom / what type of contraception to use		
Uptake of DREAMS interventions	• Invited to participate / enrolled in DREAMS • Received at least one DREAMS intervention • Received multiple intervention categories in the DREAMS core package • Received DREAMS primary or secondary interventions (depending on age and need)		Intervention sites (versus comparison sites)
			Within intervention sites, measures of individual uptake will include: • Received at least one DREAMS intervention • Received DREAMS package for key populations (‘KP_Prev’^c^) • Received KP_Prev + educational subsidies or vocational training • Received KP_Prev + PrEP

^a^In South Africa and Gem, Kenya, HIV incidence will be estimated from the larger population-level studies (see Table 2), for adequate statistical power (see ‘Sample Sizes’ below)

^b^ Age at enrolment, to be followed over two years

^c^‘KP_Prev’ is the PEPFAR indicator used to measure DREAMS package for key populations and includes condom promotion, HIV testing services, and social asset building [22]

‘Mediators of change’ (Fig. 1 and Table 3) will be measured at the individual level, representing the DREAMS Partnership’s commitment that the interventions will increase determination, resilience, empowerment, social assets, and personal safety among AGYW.

### Analysis plan

### Analysis of primary outcome

In the South African and western Kenyan sites, we will analyze population level change in directly-observed HIV incidence over time, with all data available from the HDSS sero-surveys, making comparisons among three calendar time periods as follows:

- Pre-DREAMS roll-out: the 5–10 years prior to DREAMS (up to and including 2015 in South Africa and 2016 in Gem, Kenya [see ‘Sample Sizes’ below for details])
- During early DREAMS implementation: 2016
- Post-DREAMS: 2017–2019 (rolled out)

Our main comparison will be between the post-DREAMS time period, and the two earlier time periods.

In Zimbabwe, we will compare HIV incidence between sites where DREAMS+PrEP has and has not been implemented, over two years of follow-up. In the absence of randomization, the analysis will adjust for known individual-level determinants of HIV incidence.

#### Analysis of secondary outcomes

Secondary outcomes will be captured via the HDSS in the three surveillance sites (Table 2) as well as via the cohorts of young women in all four settings (Table 3). We will analyze population and individual level change, respectively, over time in these outcomes, using the calendar time periods described above for HIV incidence.

#### Analysis of causal pathways

To explore whether the hypothesized “mediators of change” lie on the causal pathway between the DREAMS interventions and HIV-related (secondary) outcomes (Table 2), longitudinal data collected from the nested AGYW cohorts at three time points over two years will be used (enrolment; 12 months; 24 months). The causal analysis will involve four main steps:

1. Analysis of whether uptake of DREAMS interventions is related to an improvement in the “mediators of change”, between enrolment and follow-up at 12 and 24 months
2. Analysis of whether uptake of DREAMS interventions is related to “lower-risk” sexual behaviour, social protections, and biological protections, at follow-up at 12 and 24 months
3. Analysis of whether improved levels of the “mediators of change” are related to “lower-risk” sexual behavior, social protections, and biological protections, at enrolment and during follow-up
4. Causal mediation analysis of the effect of DREAMS interventions on secondary outcomes (biological, behavioral, social), i.e., the extent to which any effect of DREAMS interventions on secondary outcomes after 12 and 24 months of follow-up is achieved through their effect on the “mediating” variables.

These analyses will adjust for important confounding variables measured at enrolment (for example, household socio-economic position) and an AGYW’s “propensity to receive” DREAMS interventions. This is because the criteria used by DREAMS implementing partners to select AGYW who will be invited to participate in the programme are also likely to influence the outcomes that are to be measured, i.e., they may be risk factors for HIV incidence and the secondary outcomes, and so may confound observed associations between uptake of interventions and these outcomes. For example, Implementing Partners are prioritising girls considered to have the highest risk of HIV infection (such as, those who are living in relatively poor households, are orphans, are out of school, or are young mothers, as identified via the ‘Girl Roster’ enumeration exercise and by community-based organisations) [19]. The characteristics that predict exposure to DREAMS will be identified using HDSS data, and the information across these characteristics will be synthesized into a single “propensity to be exposed to DREAMS” score (equivalent to an estimated probability of exposure to DREAMS, and taking values between 0 and 1), and AGYW will be categorized (stratified) into 4–5 groups on the basis of their propensity score. The association between uptake of DREAMs interventions and socio-economic and behavioural outcomes will be adjusted for the propensity score (in categories) as well as the individual characteristics that are the most important confounding variables.

#### Qualitative and process evaluation data

Analysis of the concurrent process evaluation data will follow the UK Medical Research Council guidance for process evaluation of complex interventions [18]. Data collected using the range of different methods, detailed above, will be carefully integrated to address the following process evaluation questions:

- How is delivery of DREAMS achieved and what is actually delivered? (Implementation)
- How does the delivered intervention produce change? (Mechanisms of impact)
- How does context affect implementation and outcomes? (Context)

The mechanisms to be scrutinised include increased demand for (awareness and acceptability), supply of (accessibility and availability), and adherence to (ongoing adoption) the interventions in the DREAMS core package, as per the HIV prevention cascade framework, to achieve coverage among the target populations [11].

#### Sample sizes

**Fig. 3:**
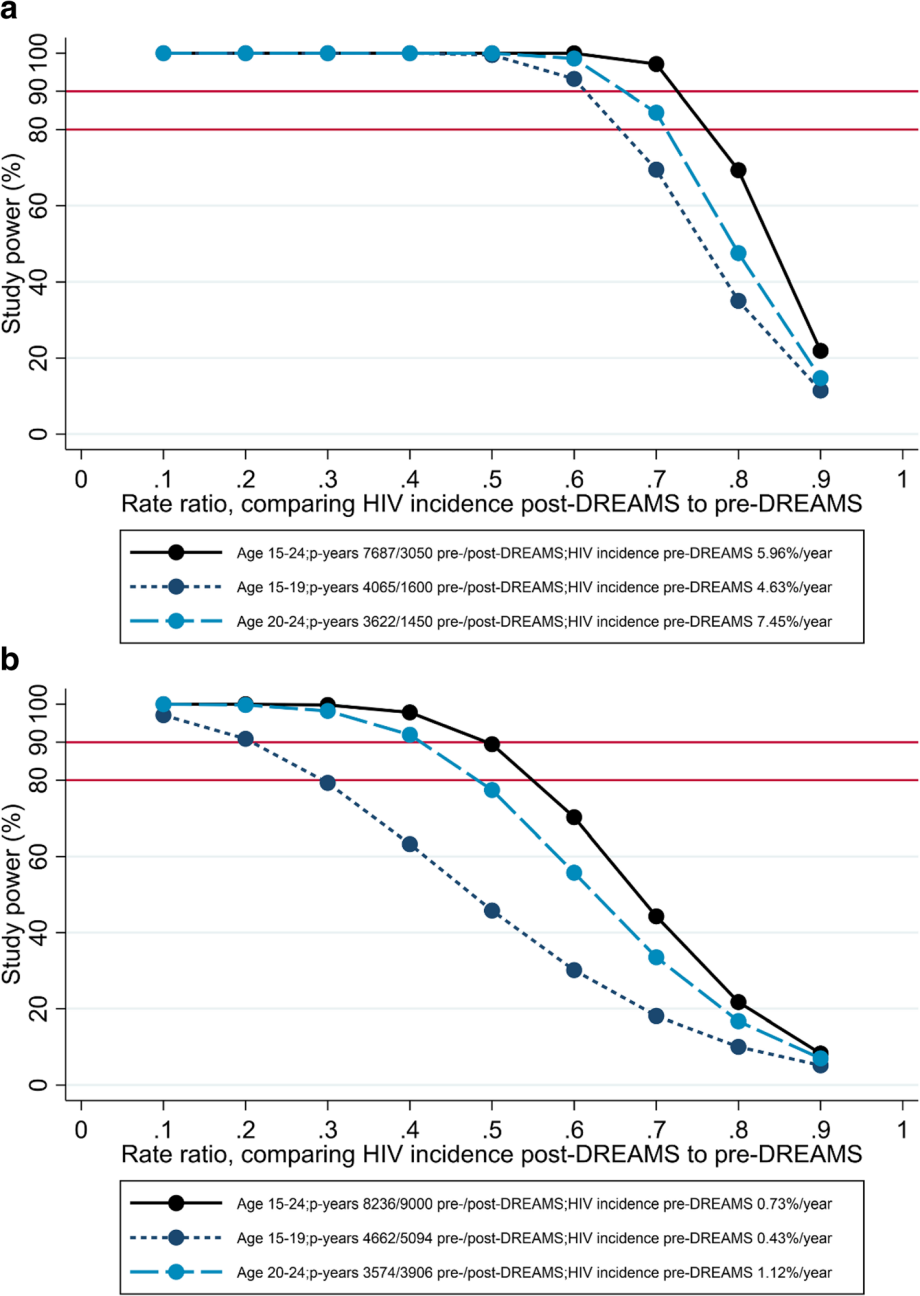
Study power for comparing HIV incidence between post-DREAMS and pre-DREAMS time periods in (**a**) uMkhanyakude, South Africa and (**b**) Gem, Kenya

HIV incidence - the primary endpoint for the impact evaluation - will be measured using HDSS data in uMkhanyakude and Gem, and data from the cohorts of young women who sell sex in Zimbabwe. In uMkhanyakude, HIV incidence was ~ 6 per 100 person-years among AGYW aged 15–24 years old during 2011–2015, and ~ 4.6 and ~ 7.5 per 100 person-years among those aged 15–19 and 20–24 years respectively, based on a total of 7687 person-years of follow-up. Assuming, conservatively, that there will be ~ 3000 person-years of follow-up during 2017–2019 (40% of 7687), [20] then study power is > 90% to show an overall reduction in HIV incidence of 30, and > 90% in sub-group analysis of AGYW aged 15–19 and 20–24 years to show a 40% reduction in HIV incidence (Fig. 3a). In Gem, western Kenya, HIV incidence was ~ 0.7 per 100 person-years among AGYW aged 15–24 years during 2011–2016, based on a total of 8236 person-years of follow-up [21]. (Whereas sero-surveys are conducted annually in uMkhanyakude, they are less frequent in Gem: data are available from three sero-surveys in Gem between 2011 and 2016. Sero-conversions observed during this period, including those estimated from the 2016 survey, will be considered ‘pre-DREAMS’ because they are unlikely to be influenced by DREAMS by this early stage of implementation.) During 2017–2019, all AGYW in Gem will be sought for participation in the HDSS; with a participation rate of 70% in each year, and an annual out-migration rate of 20%, it is estimated that there will be ~ 9000 person-years of follow-up during 2016–2019. Study power is low to show a change in HIV incidence in sub-group analyses of AGYW aged 15–19 and 20–24 years, but ~ 80% to show an overall reduction of 45, and 90% to show an overall reduction of 50% in HIV incidence (Fig. 3b).

The same sample sizes will also allow us to detect meaningful change in secondary outcomes that are more common among AGYW than HIV incidence, including knowledge of HIV status and use of condoms (Additional file 1: Table S2). Secondary outcomes will also be measured at the population level for men including the proportion of males who know their HIV status, uptake of voluntary male medical circumcision among HIV-negative males, and uptake of anti-retroviral therapy among HIV-positive males (Additional file 1: Table S3).

For the nested cohorts in the three HDSS sites, selected from the HDSS sampling frames in South Africa and Western Kenya, a minimum of 400 girls in each of the 13–17 and 18–22 year age groups will allow us to analyze causal relationships between key mediators and key outcomes (Additional file 1: Table S4), and similarly to analyze causal relationships between uptake of DREAMS interventions and key outcomes. Over-sampling by 20% will cater for non-response and loss-to-follow-up. In Nairobi, for the additional cohort of 400 younger girls aged 10–14), the sample size of 400 will allow us to explore pathways between uptake of DREAMS interventions, key mediators and age-appropriate outcomes like school completion.

In Zimbabwe, network-based recruitment will be used to enroll 18 to 24-year-old women who sell sex from intervention and comparison sites. Based on the assumption that 20% of YWSS identified through this process will test HIV-positive and 30% of HIV-negative YWSS will be lost to follow-up over 24 months, it is estimated that 1200 women from the intervention and comparison sites (2400 total) will be needed to detect a 40% reduction in HIV incidence [8]. This sample size is also sufficient to explore pathways linking DREAMS to secondary outcomes.

## Discussion

DREAMS is a direct response – probably the most ambitious yet – to the call for combinations or ‘packages’ of prevention approaches to address the multidimensional nature of HIV risk. PEPFAR and its DREAMS partners have set bold targets and allocated significant resources to urgently reduce new HIV infections. It is important to learn from these efforts, but evaluating such a multi-component programme is complex.

In the first instance, a randomised design was not possible because DREAMS sites were not selected at random, but chosen for their high burden of HIV prevalence and incidence. Furthermore, interventions in the core package cannot be rolled out at random, as implementation will begin with the interventions already in place (e.g., through pre-existing PEPFAR and national government programmes) and this is context-specific. Neither was a controlled design possible, given the numerous differences (non-comparability) across sub-national geographic units, as well as the absence of existing surveillance/data platforms in other areas, to allow for comparable data collection.

We have proposed the most rigorous design feasible in the absence of randomisation. Community-wide data platforms allow us to evaluate DREAMS in large, general populations, and provide the frameworks for randomly selected, representative samples of young people for detailed, nested studies. The range of data available (HIV, demographic, social, spatial, clinical) can be linked to maximize the range and depth of inquiry. In all settings, detailed longitudinal data will allow us to investigate pathways and explore change processes in the context of DREAMS roll-out (and minimize recall and reporting bias), and to account for a range of potential confounding variables. Historical measures of HIV incidence and other outcomes will provide baseline trend data, to help distinguish the impact of DREAMS from existing trends due to other factors.

In this study, estimates of HIV incidence will be directly observed through repeat testing and can be compared to levels of newly diagnosed infections in pregnant women tested in antenatal clinics serving the study populations, since the latter is a method by which PEPFAR will assess programme impact in DREAMS sites (e.g., through monitoring of ante-natal care testing data as part of Prevention of Mother to Child Transmission programmes) [22]. Tracking new HIV diagnoses can be a helpful complement to incidence rates, offering insight into the reach and yield of HIV testing services.

A particular challenge of this evaluation is also one of its main strengths: harmonizing across diverse settings. Each setting presents unique opportunities to deepen the understanding of AGYW’s experience of DREAMS, but coordinating the design and measures across settings is not always possible. For example, in Nairobi, we have an opportunity to understand DREAMS impact in an urban setting and to capitalize on the site’s extensive experience with young people, to track pathways through DREAMS from a very young age. In this setting, however, we will not be able to observe change in HIV incidence (except indirectly, by monitoring antenatal clinic outcomes of HIV testing). Gem offers a rural comparison to Nairobi, where we can measure the added value of DREAMS following wide-scale roll-out of anti-retroviral therapy and VMMC. In uMkhanyakude, we have an opportunity to evaluate DREAMS in a setting where HIV risk has remained persistently high and relatively few HIV prevention interventions have targeted AGYW prior to DREAMS. In Zimbabwe, no HDSS framework exists, but we will gain insight into the DREAMS+PrEP package, and understand HIV and HIV-related outcomes among an exceptionally vulnerable group of young women.

Working with existing research platforms offers infrastructure, experience and data prior to DREAMS introduction. However, it also means that data collection cannot be conducted at the same time in each site, and this must be taken into account in analyses and interpretation. Also, community sensitisation efforts are planned in each site to avoid research fatigue and maximize data quality and validity in settings with frequent and/or concurrent surveys. Furthermore, in each setting, DREAMS is delivered through different models of collaboration and implementation, and changes in implementation will occur in each setting over time. This heterogeneity and its influence on outcomes will need to be understood through careful process evaluation.

With a portfolio of evaluations in diverse settings, the sum can be greater than its parts. Learning within and across sites, we can document the role of context and adaptation in DREAMS impact, to inform replication in a range of other diverse settings. The effectiveness of the individual interventions in the DREAMS core package have been demonstrated in previous trials and evaluations. We now need to understand how they can be combined for maximum reach, scale and impact. This evaluation will investigate this in ‘real-world’, non-trial conditions, providing immediately relevant and timely lessons for future policy and programming.

## Additional file

Additional file 1: **Table S1**. Summary of primary and secondary packages of interventions in each country setting, by age where applicable. **Table S2**. Estimated sample sizes to measure change over time in secondary outcomes among AGYW. **Table S3**. Estimated sample sizes to measure change over time in key outcomes among males. **Table S4**. Estimated sample sizes to assess the causal effect of key mediators of change on secondary outcomes (via cohorts of adolescent girls and young women). (DOCX 58 kb)

## Abbreviations

AGYW: Adolescent girls and young women
AHRI: Africa Health Research Institute
APHRC: African Population and Health Research Center
ART: Anti-retroviral therapy
CDC: United States Centers for Disease Control and Prevention
CeSHHAR: Centre for Sexual Health and HIV AIDS Research
DREAMS: Determined, Resilient, Empowered, AIDS-free, Mentored, Safe lives
FGD: Focus group discussion
FSW: Female sex workers
GEAS: Global Early Adolescent Study
HDSS: Health and demographic surveillance system
HIV: Human immunodeficiency virus
HSV-2: Herpes simplex virus type 2
HTC: HIV testing and counselling
HTS: HIV testing services
IE: Impact Evaluation
IP: Implementing Partner for DREAMS
KEMRI: Kenya Medical Research Institute
KP_Prev: Number of key populations reached with HIV prevention interventions
KZN: KwaZulu-Natal
LSHTM: London School of Hygiene & Tropical Medicine
LSTM: Liverpool School of Tropical Medicine
NHLS: National Health Laboratory Services
PEP: Post-exposure prophylaxis (for HIV)
PEPFAR: The United States President’s Emergency Plan for AIDS Relief
PrEP: Pre-exposure prophylaxis (for HIV)
SRH: Sexual and reproductive health
STI: Sexually transmitted infection
UK: United Kingdom
US: United States
VMMC: Voluntary male medical circumcision
YWSS: Young women who sell sex
