# Supplementary material for: Evaluating the impact of the DREAMS partnership to reduce HIV incidence among adolescent girls and young women in four settings: a study protocol
Source: BMC Public Health. 2018 Jul 25;18:912. doi: 10.1186/s12889-018-5789-7 (PMC6060450; doi:10.1186/s12889-018-5789-7)
Supplement: Supplementary file 1 — Table S1. Summary of primary and secondary packages of interventions in each country setting, by age where applicable. Table S2. Estimated sample sizes to measure change over time in secondary outcomes among AGYW. Table S3. Estimated sample sizes to measure change over time in key outcomes among males. Table S4. Estimated sample sizes to assess the causal effect of key mediators of change on secondary outcomes (via cohorts of adolescent girls and young women). (DOCX 58 kb) [file 12889_2018_5789_MOESM1_ESM.docx]

**Table S1. Summary of primary and secondary packages of interventions in each country setting, by age where applicable**

| **Setting** | **List of constituent interventions** |
| --- | --- |
| Kenya | Primary interventions   - HIV Testing and Counselling (age 10-24) - Social asset building (age 10-24) - School-based HIV and violence prevention (age 10-24) - Financial capability training (age 10-24) - Entrepreneurship training (age 15-24) - Condom education and promotion (age 15-24) - Contraceptive method mix education and counselling (age 15-24) - PrEP education and counselling (age 18-24)   Secondary interventions (needs based)   - Families Matter Programme (age 10-14) - Educational subsidies (age 10-24) - Post violence care (age 10-24) - Cash transfers (age 15-24) - Combined socio-economic approaches (age 15-24) - Condom provision (age 15-24) - Contraceptive method mix provision (age 15-24) - PrEP provision (age 18-24) |
| South Africa | Primary interventions   - School-based HIV and violence prevention (age 10-19) - Social asset building (age 10-19) - Condoms (sexually active age 10-24) - HIV Testing and Counselling (sexually active age 10-24) - Sexual and reproductive health (sexually active age 10-24)   Secondary interventions (needs based)   - Material support e.g. cash transfers and educational subsidies (age 10-19) - Post violence care (age 10-24) - Combined socio-economic approaches (age 10-24) - PrEP (age 18-24) |
| Zimbabwe* | Primary interventions   - HIV Testing and Counselling (age 15-24) - Condom promotion and provision (age 15-24) - Social asset building (age 15-24)   Secondary interventions (needs based)   - Disability assessment (age 15-24) - Education support or combination socio-economic approaches (age 15-24) - Family planning services (age 15-24) - Gender based violence response (age 15-24) - Health services (other STIs) (age 15-24) - PrEP (age 15-24) |

*’KP_Prev’ – PEPFAR indicator for the DREAMS package of HIV prevention services for key populations [19]

**Table S2. Estimated sample sizes to measure change over time in secondary outcomes among AGYW**

| **Key outcomes of interest**  **for AGYW** | **15-19 year old adolescent girls** | | | | | | | **20-24 year old young women** | | | | | | |
| --- | --- | --- | --- | --- | --- | --- | --- | --- | --- | --- | --- | --- | --- | --- |
|  | **Prevalence** | | | **Estimated sample sizes** | | | | **Prevalence** | | | **Estimated sample sizes** | | | |
|  | **Pre-DREAMS** | **Post-DREAMS** | **Ratio‡** | **80% power** | **90% power** | **Precision** ±**10%^†^** | **Precision**  ±**2.5%^β^** | **Pre-DREAMS** | **Post-DREAMS** | **Ratio‡** | **80% power** | **90% power** | **Precision** ±**10%^†^** | **Precision**  ±**2.5%^β^** |
| **Proportion of AGYW who know their HIV status** | 20% | 50% | 2.50 | 45 | 58 | 385 | 1537 | 30% | 60% | 2.00 | 49 | 63 | 257 | 1476 |
|  | 30% | 60% | 2.00 | 49 | 63 | 257 | 1476 | 40% | 70% | 1.75 | 49 | 63 | 165 | 1291 |
|  | 40% | 70% | 1.75 | 49 | 63 | 165 | 1291 | 50% | 80% | 1.60 | 45 | 58 | 97 | 984 |
|  | 50% | 80% | 1.60 | 45 | 58 | 97 | 984 | 60% | 90% | 1.50 | 38 | 49 | 43 | 554 |
| **Proportion of AGYW who have ever had sex** | 45% | 20% | 0.44 | 62 | 80 | 1537 | 984 | 85% | 40% | 0.47 | 22 | 27 | 577 | 1476 |
|  |  | 30% | 0.67 | 176 | 230 | 897 | 1291 |  | 50% | 0.59 | 33 | 42 | 385 | 1537 |
|  | 50% | 25% | 0.50 | 66 | 85 | 1153 | 1153 | 90% | 45% | 0.50 | 20 | 25 | 470 | 1522 |
|  | 55% | 30% | 0.55 | 68 | 88 | 897 | 1291 | 95% | 50% | 0.53 | 19 | 23 | 385 | 1537 |
| **Proportion of AGYW [aged 18-19] who first had sex aged <18 years** | 40% | 20% | 0.50 | 91 | 119 | 1537 | 984 |  |  |  |  |  |  |  |
|  |  | 30% | 0.75 | 376 | 496 | 897 | 1291 |  |  |  |  |  |  |  |
|  | 50% | 30% | 0.60 | 103 | 134 | 897 | 1291 |  |  |  |  |  |  |  |
|  | 60% | 40% | 0.67 | 107 | 140 | 577 | 1476 |  |  |  |  |  |  |  |
| **Proportion of AGYW who used a condom last time they had sex, during the past 12 months** | 5% | 40% | 8.00 | 27 | 34 | 577 | 1476 | 5% | 40% | 8.00 | 27 | 34 | 577 | 1476 |
|  |  | 20% | 4.00 | 88 | 114 | 1537 | 984 |  | 20% | 4.00 | 88 | 114 | 1537 | 984 |
|  | 10% | 45% | 4.50 | 30 | 38 | 470 | 1522 | 10% | 45% | 4.50 | 30 | 38 | 470 | 1522 |
|  | 15% | 50% | 3.33 | 33 | 42 | 385 | 1537 | 15% | 50% | 3.33 | 33 | 42 | 385 | 1537 |
| **Proportion of AGYW who had >1 sexual partner during the past 12 months** | 2% | 1% | 0.50 | 2515 | 3300 | 38032 | 61 | 2% | 1% | 0.50 | 2515 | 3300 | 38032 | 61 |
|  | 5% | 2.5% | 0.50 | 984 | 1291 | 14983 | 150 | 5% | 2.5% | 0.50 | 984 | 1291 | 14983 | 150 |
|  | 10% | 5% | 0.50 | 474 | 621 | 7300 | 292 | 10% | 5% | 0.50 | 474 | 621 | 7300 | 292 |
|  | 15% | 7.5% | 0.50 | 304 | 398 | 4738 | 427 | 15% | 7.5% | 0.50 | 304 | 398 | 4738 | 427 |
| **Proportion of AGYW [aged 18-19] pregnant before age 18** | 15% | 7.5% | 0.50 | 304 | 398 | 4738 | 427 |  |  |  |  |  |  |  |
|  |  | 10% | 0.67 | 726 | 957 | 3458 | 554 |  |  |  |  |  |  |  |
|  | 20% | 10% | 0.50 | 219 | 286 | 3458 | 554 |  |  |  |  |  |  |  |
|  | 25% | 12.5% | 0.50 | 168 | 219 | 2690 | 673 |  |  |  |  |  |  |  |
| **Proportion of AGYW who had a sexual partner >5 years older/younger in the past 12m** | 20% | 10% | 0.50 | 219 | 286 | 3458 | 554 | 50% | 25% | 0.50 | 66 | 85 | 1153 | 1153 |
|  | 30% | 15% | 0.50 | 134 | 174 | 2177 | 784 | 60% | 30% | 0.50 | 49 | 63 | 897 | 1291 |
|  | 40% | 20% | 0.50 | 91 | 119 | 1537 | 984 | 70% | 35% | 0.50 | 37 | 47 | 714 | 1399 |
| **Proportion of AGYW who have ever experienced sexual abuse/violence** | 10% | 5% | 0.50 | 474 | 621 | 7300 |  | 10% | 5% | 0.50 | 474 | 621 | 7300 |  |
|  | 15% | 7.5% | 0.50 | 304 | 398 | 4738 |  | 20% | 10% | 0.50 | 219 | 286 | 3458 |  |
|  | 20% | 10% | 0.50 | 219 | 286 | 3458 |  | 30% | 15% | 0.50 | 134 | 174 | 2177 |  |
| **Proportion of AGYW [aged 18-19] who are in or have completed school** | 30% | 50% | 1.67 | 103 | 134 | 385 |  |  |  |  |  |  |  |  |
|  |  | 40% | 1.33 | 376 | 496 | 577 |  |  |  |  |  |  |  |  |
|  | 40% | 60% | 1.50 | 107 | 140 | 257 |  |  |  |  |  |  |  |  |
|  | 50% | 70% | 1.400 | 103 | 134 | 42 |  |  |  |  |  |  |  |  |

**‡**Post-DREAMS vs pre-DREAMS prevalence

**†**Sample size required to estimate the post-DREAMS prevalence to within ±10% of the point estimate (“relative” precision)

^β^Sample size required to estimate the post-DREAMS prevalence to within ±2.5 percentage points (“absolute” precision)

**Footnote**: The sample sizes refer to the numbers required both pre-DREAMS and again post-DREAMS.

**Table S3. Estimated sample sizes to measure change over time in key outcomes among males**

| **Outcome of interest for males** | **Age** | **Prevalence** | |  | **Estimated sample sizes** | | | |
| --- | --- | --- | --- | --- | --- | --- | --- | --- |
|  |  | **Pre-DREAMS** | **Post-DREAMS** | **Ratio‡** | **80% power** | **90% power** | **Precision** ±**10%^†^** | **Precision**  ±**2.5%^β^** |
| **Proportion of males who know their HIV status** | 15-24 | 30% | 60% | 2.00 | 49 | 63 | 257 | 1476 |
|  |  |  | 70% | 2.33 | 29 | 36 | 165 | 1291 |
|  |  | 40% | 70% | 1.75 | 49 | 63 | 165 | 1291 |
|  |  |  | 80% | 2.00 | 28 | 35 | 97 | 984 |
|  |  | 50% | 80% | 1.60 | 45 | 58 | 97 | 984 |
|  |  |  | 90% | 1.80 | 25 | 31 | 43 | 554 |
|  | 25-34 | 40% | 65% | 1.63 | 70 | 90 | 207 | 1399 |
|  |  |  | 75% | 1.88 | 36 | 46 | 129 | 1153 |
|  |  | 50% | 75% | 1.50 | 66 | 85 | 129 | 1153 |
|  |  |  | 85% | 1.70 | 33 | 42 | 68 | 784 |
|  |  | 60% | 80% | 1.33 | 91 | 119 | 97 | 984 |
|  |  |  | 90% | 1.50 | 38 | 49 | 43 | 554 |
|  | 35-44 | 40% | 65% | 1.63 | 70 | 90 | 207 | 1399 |
|  |  |  | 75% | 1.88 | 36 | 46 | 129 | 1153 |
|  |  | 50% | 75% | 1.50 | 66 | 85 | 129 | 1153 |
|  |  |  | 85% | 1.70 | 33 | 42 | 68 | 784 |
|  |  | 60% | 80% | 1.33 | 91 | 119 | 97 | 984 |
|  |  |  | 90% | 1.50 | 38 | 49 | 43 | 554 |
|  | 45-54 | 40% | 65% | 1.63 | 70 | 90 | 207 | 1399 |
|  |  |  | 75% | 1.88 | 36 | 46 | 129 | 1153 |
|  |  | 50% | 75% | 1.50 | 66 | 85 | 129 | 1153 |
|  |  |  | 85% | 1.70 | 33 | 42 | 68 | 784 |
|  |  | 60% | 80% | 1.33 | 91 | 119 | 97 | 984 |
|  |  |  | 90% | 1.50 | 38 | 49 | 43 | 554 |
|  | 25-54 | 40% | 65% | 1.63 | 70 | 90 | 207 | 1399 |
|  |  |  | 75% | 1.88 | 36 | 46 | 129 | 1153 |
|  |  | 50% | 75% | 1.50 | 66 | 85 | 129 | 1153 |
|  |  |  | 85% | 1.70 | 33 | 42 | 68 | 784 |
|  |  | 60% | 80% | 1.33 | 91 | 119 | 97 | 984 |
|  |  |  | 90% | 1.50 | 38 | 49 | 43 | 554 |
| **Voluntary male medical circumcision (VMMC) among HIV- males** | 15-24 | 20% | 50% | 2.50 | 45 | 58 | 385 | 1537 |
|  |  |  | 60% | 3.00 | 28 | 35 | 257 | 1476 |
|  |  | 30% | 50% | 1.67 | 103 | 134 | 385 | 1537 |
|  |  |  | 60% | 2.00 | 49 | 63 | 257 | 1476 |
|  |  | 40% | 60% | 1.50 | 107 | 140 | 257 | 1476 |
|  |  |  | 70% | 1.75 | 49 | 63 | 165 | 1291 |
|  |  | 50% | 70% | 1.40 | 103 | 134 | 165 | 1291 |
|  |  |  | 80% | 1.60 | 45 | 58 | 97 | 984 |
|  | 25-34 | 30% | 50% | 1.67 | 103 | 134 | 385 | 1537 |
|  |  |  | 60% | 2.00 | 49 | 63 | 257 | 1476 |
|  |  | 40% | 60% | 1.50 | 107 | 140 | 257 | 1476 |
|  |  |  | 70% | 1.75 | 49 | 63 | 165 | 1291 |
|  |  | 50% | 70% | 1.40 | 103 | 134 | 165 | 1291 |
|  |  |  | 80% | 1.60 | 45 | 58 | 97 | 984 |
|  | 35-44 | 30% | 50% | 1.67 | 103 | 134 | 385 | 1537 |
|  |  |  | 60% | 2.00 | 49 | 63 | 257 | 1476 |
|  |  | 40% | 60% | 1.50 | 107 | 140 | 257 | 1476 |
|  |  |  | 70% | 1.75 | 49 | 63 | 165 | 1291 |
|  |  | 50% | 70% | 1.40 | 103 | 134 | 165 | 1291 |
|  |  |  | 80% | 1.60 | 45 | 58 | 97 | 984 |
|  | 45-54 | 30% | 50% | 1.67 | 103 | 134 | 385 | 1537 |
|  |  |  | 60% | 2.00 | 49 | 63 | 257 | 1476 |
|  |  | 40% | 60% | 1.50 | 107 | 140 | 257 | 1476 |
|  |  |  | 70% | 1.75 | 49 | 63 | 165 | 1291 |
|  |  | 50% | 70% | 1.40 | 103 | 134 | 165 | 1291 |
|  |  |  | 80% | 1.60 | 45 | 58 | 97 | 984 |
|  | 25-54 | 30% | 50% | 1.67 | 103 | 134 | 385 | 1537 |
|  |  |  | 60% | 2.00 | 49 | 63 | 257 | 1476 |
|  |  | 40% | 60% | 1.50 | 107 | 140 | 257 | 1476 |
|  |  |  | 70% | 1.75 | 49 | 63 | 165 | 1291 |
|  |  | 50% | 70% | 1.40 | 103 | 134 | 165 | 1291 |
|  |  |  | 80% | 1.60 | 45 | 58 | 97 | 984 |
| **Uptake of ART among HIV+ males in past 12 months** | 15-24 | 20% | 50% | 2.50 | 45 | 58 | 385 | 1537 |
|  |  |  | 60% | 3.00 | 28 | 35 | 257 | 1476 |
|  |  | 30% | 60% | 2.00 | 49 | 63 | 257 | 1476 |
|  |  |  | 70% | 2.33 | 29 | 36 | 165 | 1291 |
|  |  | 40% | 70% | 1.75 | 49 | 63 | 165 | 1291 |
|  |  |  | 80% | 2.00 | 28 | 35 | 97 | 984 |
|  | 25-34 | 30% | 60% | 2.00 | 49 | 63 | 257 | 1476 |
|  |  |  | 70% | 2.33 | 29 | 36 | 165 | 1291 |
|  |  | 40% | 65% | 1.63 | 70 | 90 | 207 | 1399 |
|  |  |  | 75% | 1.88 | 36 | 46 | 129 | 1153 |
|  |  | 50% | 70% | 1.40 | 103 | 134 | 165 | 1291 |
|  |  |  | 80% | 1.60 | 45 | 58 | 97 | 984 |
|  | 35-44 | 30% | 60% | 2.00 | 49 | 63 | 257 | 1476 |
|  |  |  | 70% | 2.33 | 29 | 36 | 165 | 1291 |
|  |  | 40% | 65% | 1.63 | 70 | 90 | 207 | 1399 |
|  |  |  | 75% | 1.88 | 36 | 46 | 129 | 1153 |
|  |  | 50% | 70% | 1.40 | 103 | 134 | 165 | 1291 |
|  |  |  | 80% | 1.60 | 45 | 58 | 97 | 984 |
|  | 45-54 | 30% | 60% | 2.00 | 49 | 63 | 257 | 1476 |
|  |  |  | 70% | 2.33 | 29 | 36 | 165 | 1291 |
|  |  | 40% | 65% | 1.63 | 70 | 90 | 207 | 1399 |
|  |  |  | 75% | 1.88 | 36 | 46 | 129 | 1153 |
|  |  | 50% | 70% | 1.40 | 103 | 134 | 165 | 1291 |
|  |  |  | 80% | 1.60 | 45 | 58 | 97 | 984 |
|  | 25-54 | 30% | 60% | 2.00 | 49 | 63 | 257 | 1476 |
|  |  |  | 70% | 2.33 | 29 | 36 | 165 | 1291 |
|  |  | 40% | 65% | 1.63 | 70 | 90 | 207 | 1399 |
|  |  |  | 75% | 1.88 | 36 | 46 | 129 | 1153 |
|  |  | 50% | 70% | 1.40 | 103 | 134 | 165 | 1291 |
|  |  |  | 80% | 1.60 | 45 | 58 | 97 | 984 |

t-DREAMS vs pre-DREAMS prevalence.

**†**Sample size required to estimate the post-DREAMS prevalence to within ±10% of the point estimate (“relative” precision)

^β^Sample size required to estimate the post-DREAMS prevalence to within ±2.5 percentage points (“absolute” precision)

**Footnote**: The sample sizes for VMMC among HIV- males refer to the numbers required for analysis however the prevalence of HIV needs to be taken into account for the actual number needed to sample. Similarly, the figures for uptake of ART among HIV+ males represent the numbers required for analysis before accounting for HIV prevalence. If HIV prevalence is 10% the number needed to sample size will be 10 times larger since only 10% of those sampled would be HIV+; if prevalence is 20% the actual number needed to sample will be 5 times larger.

***Table S4. Estimated sample sizes to assess the causal effect of key ‘mediators of change’ on secondary outcomes (via cohorts of AGYW)***

| **Mediator present at baseline** | | **Prevalence of outcome at 24 months**  **by baseline mediator category** | | **Prevalence ratio** | **Sample size**† | |
| --- | --- | --- | --- | --- | --- | --- |
| **no** | **yes** | **Mediator=no** | **Mediator=yes** | **[yes vs no]** | **80% power** | **90% power** |
| 90% | 10% | 10% | 7.5% | 0.75 | 15001 | 19523 |
|  |  |  | 5% | 0.50 | 3536 | 4444 |
|  |  |  | 2.5% | 0.25 | 1447 | 1736 |
|  |  | 30% | 20% | 0.67 | 2244 | 2899 |
|  |  |  | 15% | 0.50 | 973 | 1229 |
|  |  |  | 10% | 0.33 | 528 | 647 |
|  |  | 50% | 30% | 0.60 | 721 | 928 |
|  |  |  | 20% | 0.40 | 317 | 397 |
|  |  |  | 10% | 0.20 | 171 | 204 |
|  |  | 70% | 40% | 0.57 | 321 | 416 |
|  |  |  | 30% | 0.43 | 184 | 234 |
|  |  |  | 20% | 0.29 | 119 | 146 |
|  |  | 90% | 60% | 0.67 | 219 | 294 |
|  |  |  | 50% | 0.56 | 137 | 182 |
|  |  |  | 40% | 0.44 | 94 | 123 |
| 70% | 30% | 10% | 7.5% | 0.75 | 6321 | 8301 |
|  |  |  | 5% | 0.50 | 1469 | 1886 |
|  |  |  | 2.5% | 0.25 | 598 | 744 |
|  |  | 30% | 20% | 0.67 | 949 | 1238 |
|  |  |  | 15% | 0.50 | 411 | 526 |
|  |  |  | 10% | 0.33 | 223 | 279 |
|  |  | 50% | 30% | 0.60 | 311 | 401 |
|  |  |  | 20% | 0.40 | 138 | 174 |
|  |  |  | 10% | 0.20 | 76 | 92 |
|  |  | 70% | 40% | 0.57 | 144 | 186 |
|  |  |  | 30% | 0.43 | 86 | 107 |
|  |  |  | 20% | 0.29 | 54 | 67 |
|  |  | 90% | 60% | 0.67 | 107 | 138 |
|  |  |  | 50% | 0.56 | 69 | 88 |
|  |  |  | 40% | 0.44 | 49 | 62 |
| 50% | 50% | 10% | 7.5% | 0.75 | 5211 | 6906 |
|  |  |  | 5% | 0.50 | 1186 | 1553 |
|  |  |  | 2.5% | 0.25 | 473 | 608 |
|  |  | 30% | 20% | 0.67 | 783 | 1031 |
|  |  |  | 15% | 0.50 | 336 | 436 |
|  |  |  | 10% | 0.33 | 181 | 231 |
|  |  | 50% | 30% | 0.60 | 258 | 336 |
|  |  |  | 20% | 0.40 | 113 | 146 |
|  |  |  | 10% | 0.20 | 63 | 78 |
|  |  | 70% | 40% | 0.57 | 123 | 158 |
|  |  |  | 30% | 0.43 | 73 | 91 |
|  |  |  | 20% | 0.29 | 48 | 58 |
|  |  | 90% | 60% | 0.67 | 96 | 123 |
|  |  |  | 50% | 0.56 | 63 | 78 |
|  |  |  | 40% | 0.44 | 43 | 53 |
| 30% | 70% | 10% | 7.5% | 0.75 | 6076 | 8131 |
|  |  |  | 5% | 0.50 | 1343 | 1797 |
|  |  |  | 2.5% | 0.25 | 513 | 684 |
|  |  | 30% | 20% | 0.67 | 913 | 1213 |
|  |  |  | 15% | 0.50 | 384 | 509 |
|  |  |  | 10% | 0.33 | 201 | 268 |
|  |  | 50% | 30% | 0.60 | 306 | 397 |
|  |  |  | 20% | 0.40 | 131 | 172 |
|  |  |  | 10% | 0.20 | 72 | 88 |
|  |  | 70% | 40% | 0.57 | 147 | 188 |
|  |  |  | 30% | 0.43 | 84 | 109 |
|  |  |  | 20% | 0.29 | 56 | 68 |
|  |  | 90% | 60% | 0.67 | 122 | 147 |
|  |  |  | 50% | 0.56 | 76 | 93 |
|  |  |  | 40% | 0.44 | 56 | 63 |
| 10% | 90% | 10% | 7.5% | 0.75 | 13863 | 18726 |
|  |  |  | 5% | 0.50 | 2951 | 4038 |
|  |  |  | 2.5% | 0.25 | 1051 | 1463 |
|  |  | 30% | 20% | 0.67 | 2076 | 2788 |
|  |  |  | 15% | 0.50 | 851 | 1151 |
|  |  |  | 10% | 0.33 | 426 | 576 |
|  |  | 50% | 30% | 0.60 | 688 | 913 |
|  |  |  | 20% | 0.40 | 288 | 376 |
|  |  |  | 10% | 0.20 | 138 | 188 |
|  |  | 70% | 40% | 0.57 | 338 | 426 |
|  |  |  | 30% | 0.43 | 188 | 238 |
|  |  |  | 20% | 0.29 | 113 | 151 |
|  |  | 90% | 60% | 0.67 | 276 | 338 |
|  |  |  | 50% | 0.56 | 176 | 213 |
|  |  |  | 40% | 0.44 | 126 | 138 |

†Figures inflated by dividing by 0.8 in order to allow for 20% loss to follow-up
